# Development of a Microfluidic Platform for Trace Lipid Analysis

**DOI:** 10.3390/metabo11030130

**Published:** 2021-02-24

**Authors:** Andrew Davic, Michael Cascio

**Affiliations:** Department of Chemistry and Biochemistry, Duquesne University, Pittsburgh, PA 15282, USA; adavic@ppg.com

**Keywords:** primary fatty acid amides, microfluidics, laser induced fluorescence, bioactive lipids

## Abstract

The inherent trace quantity of primary fatty acid amides found in biological systems presents challenges for analytical analysis and quantitation, requiring a highly sensitive detection system. The use of microfluidics provides a green sample preparation and analysis technique through small-volume fluidic flow through micron-sized channels embedded in a polydimethylsiloxane (PDMS) device. Microfluidics provides the potential of having a micro total analysis system where chromatographic separation, fluorescent tagging reactions, and detection are accomplished with no added sample handling. This study describes the development and the optimization of a microfluidic-laser induced fluorescence (LIF) analysis and detection system that can be used for the detection of ultra-trace levels of fluorescently tagged primary fatty acid amines. A PDMS microfluidic device was designed and fabricated to incorporate droplet-based flow. Droplet microfluidics have enabled on-chip fluorescent tagging reactions to be performed quickly and efficiently, with no additional sample handling. An optimized LIF optical detection system provided fluorescently tagged primary fatty acid amine detection at sub-fmol levels (436 amol). The use of this LIF detection provides unparalleled sensitivity, with detection limits several orders of magnitude lower than currently employed LC-MS techniques, and might be easily adapted for use as a complementary quantification platform for parallel MS-based omics studies.

## 1. Introduction

Primary fatty acid amides (PFAMs) are a subclass of fatty acyls important in nervous system signaling, receptor binding, and numerous other physiological roles [1]. Structurally, these bioactive lipids contain a carboxamide head group and an acyl tail varying in both length and unsaturation. Palmitamide (C16:0), palmitoleamide (C16:1^9^), oleamide (C18:1^9-cis^), elaidamide (C18:1^9-trans^), and linoleamide (C18:2^9,12^) were the first PFAMs to be identified in 1989 from luteal phase plasma [2]. Oleamide is widely studied due to its abundance of physiological roles and has been found (along with erucamide [C22:1^13^]) in the cerebrospinal fluid of sleep deprived cats [3,4,5]. In addition to sleep induction, systematic administration of oleamide affects vasorelaxation, decreased locomotor activity, and decreased body temperature [6,7,8]. Although not classified as an endocannabinoid, oleamide displays characteristic properties such as analgesia, increased appetite, and antianxiety upon high concentration binding to the cannabinoid receptors [9,10]. Oleamide has been shown to be highly selective in potentiating the action of serotonin on serotonin receptors (5-HT_2A_ and 5-HT_2C_), which produces long-lasting inhibition of GABA_A_ receptor function and thereby affects alertness, sleep, and mood [11,12,13,14]. It has also proven to be effective at blocking gap junction communication between rat glial cells while not affecting intercellular calcium signaling [15,16]. Other highly characterized PFAMs include erucamide, which modulates water balance and stimulates angiogenesis [17,18,19], linoleamide, which induces increases in Ca^2+^ concentrations in Madin-Darby Canine Kidney (MDCK) tubular cells, aiding in initiation and regulation of many cell functions [20,21,22], and anandamide (C20:4^5,8,11,14^), which acts as a endogenous ligand for cannabinoid receptors [23,24,25].

The vast array of physiological properties exhibited by PFAMs makes their study attractive in the realm of disease-state biomarker research, however, their endogenous low (nM) concentrations present analysis and detection challenges. PFAMs have been found in plasma, cerebral spinal fluid, neuroblastoma cells, and neuronal tissue [2,3,23,26]. Initial discovery of PFAMs utilized nuclear magnetic resonance spectroscopy; however, current work more commonly involves coupling gas chromatography (GC) or high performance liquid chromatography (HPLC) for separation with mass spectrometry (MS) for sensitive detection and structural identification of endogenous or derivatized analyte [2,3,4,5,17,23,24,26,27,28,29,30,31,32,33]. MS detection, although widely employed and of great benefit, results in limits of detection and quantitation on the periphery of endogenous PFAM concentrations, thus a complementary methodology for sensitive detection is required. The potential sensitivity of laser induced fluorescence (LIF) makes this method an attractive alternative detection system.

The use of fluorescent labeling coupled with LIF detection presents the theoretical ability to approach single molecule detection using a fluorophore of high quantum yield versus the relatively moderate ionization efficiency, which can be a limiting factor in MS-based detection systems. In addition, LIF detection can also provide information regarding relative concentrations of comparative chemical species, a benefit not afforded by MS, which only allows direct comparison between isotopically labeled chemical isomers that ionize equally. In the case of PFAMs, the amide must first be converted to its conjugate amine as the carbonyl of the amide causes delocalization of electron density, hindering the fluorescent reaction [34]. To achieve maximal fluorescent labeling efficiency, a microfluidic platform can be utilized. Microfluidics is the general label given to the technology of systems that process or manipulate small volumes (10^−9^ to 10^−18^ L) of fluids by utilizing embedded channels with internal diameters on the micrometer scale [35]. Microchip fabrication can be performed quickly, safely, and economically via soft lithography using compounds such as polydimethylsiloxane (PDMS) [36]. An inherent obstacle faced while performing microfluidic reactions on-chip is mixing efficiency, as laminar flow dominates the system at the micro-scale due to the low Reynolds number (Re < 2300). In laminar flow, the major form of mixing that occurs is diffusion between multiple streamlines, which is highly ineffective. Segmented flow microfluidics presents the ability to conduct highly efficient reactions on-chip entirely within small volume (nL) microdroplets. This may be performed by introducing at least two immiscible phases into the micro-channels via pressure-driven flow. One phase consists of a carrier fluid (perfluorodecalin, gas, etc.), which is continuous and encapsulates the second phase (analyte and all reaction-dependent solutions) into segmented nanodroplets [37]. Highly efficient mixing of nanodroplets relies on the repeated stretching and folding of the intra-droplet fluidic layers until these layers become increasingly thin to the point where inter-layer diffusion becomes rapid [38,39]. The mixing process, known as chaotic advection, drastically reduces reaction time while improving reaction efficiency largely due to the significantly reduced mean free path as well as the absence of diffusion outside of the nanodroplet [40]. As the droplets flow through the microchannels, frictional forces introduced via contact with the channel walls cause internal recirculation of the droplet, enhancing mixing. Implementing winding “S” shaped channels with several turns also enhances mixing by reorienting the droplet’s internal fluidic layers in the direction of droplet movement [40,41,42]. 

Herein, we describe our efforts to develop the methodologies of droplet-based microfluidics to enhance the fluorescent labeling of derivatized PFAMs. The use of µHPLC with single photon counting detection enables an in-line micro total analysis system (µTAS) with detection limits sufficient for investigation of endogenous PFAMs. Significantly, this approach provides a sensitive, quantifiable method more sensitive than typical MS-based studies, thus it can provide a complementary isotope-independent adjunct to discovery-based MS studies.

## 2. Results

### 2.1. Fluorescent Tagging of Primary Amines

**Figure 1:**
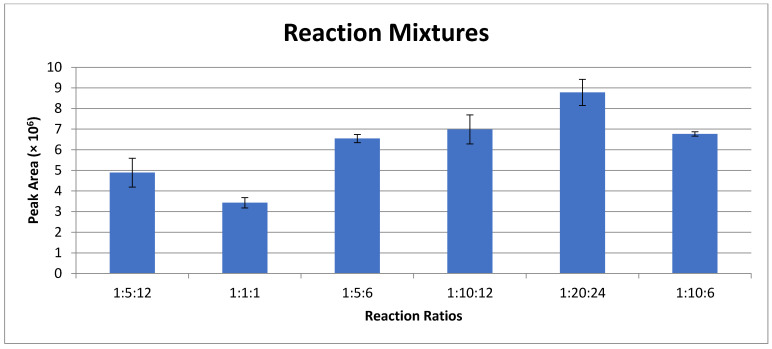
Reaction mixtures as a function of reactants stoichiometry. All solutions were prepared with reactants added in equimolar concentrations following the format of decylamine:potassium cyanide (KCN):naphthalene-2,3-dicarboxaldehyde (NDA) by volume. Average peak area of fluorescently tagged decylamine diluted to a concentration of 1 µM is shown for each reaction mixture, with standard deviations depicted (*n* = 3).

**Figure 2:**
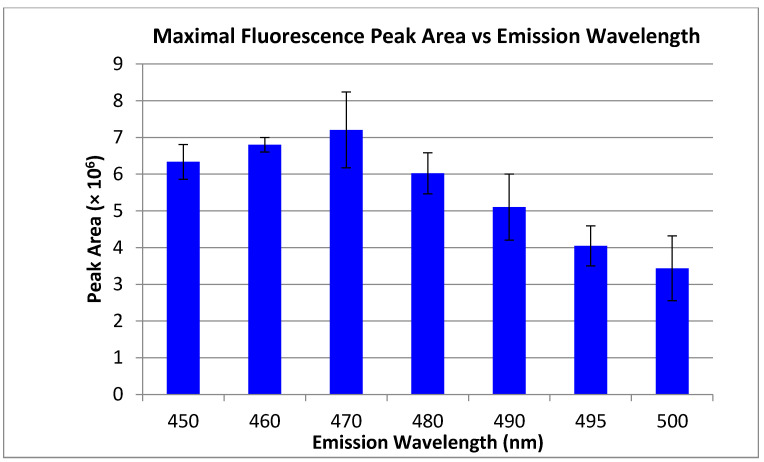
Average HPLC peak areas are shown at varying intervals of fluorescence emission wavelength. Dodecylamine was fluorescently tagged with NDA and diluted to a concentration of 1 µM. Fluorescence emission wavelengths ranged from 450 to 500 nm with the excitation wavelength held at 405 nm. Standard deviations are shown (*n* = 3).

**Figure 3:**
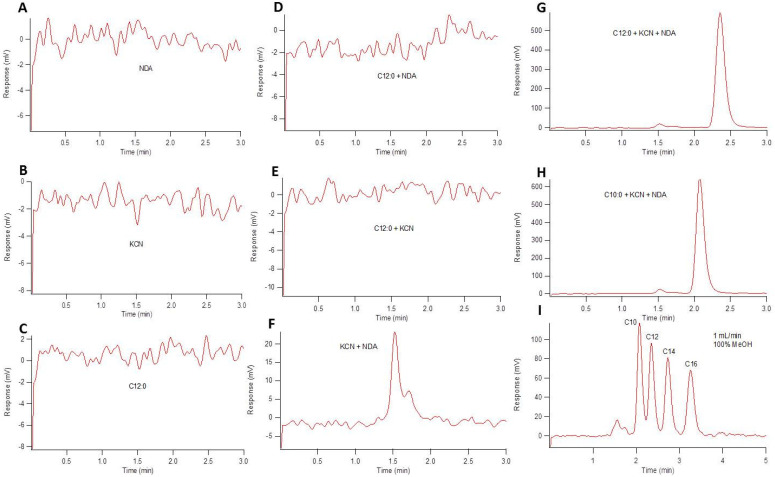
HPLC chromatograms showing native fluorescence of reactants and varying reactant mixtures. All reactants were prepared to 100 µM and incubated at 50 °C for 45 min. For mixtures, 1:10:12 (*v*/*v*/*v*) equimolar amine:KCN:NDA reaction conditions were used. (**A**–**C**) show fluorescence chromatograms of NDA, KCN, and dodecylamine, respectively. (**D**,**E**) show dodecylamine reacted with NDA and dodecylamine reacted with KCN, respectively. (**F**) shows the fluorescence response of KCN and NDA reacted with one another. (**G**,**H**) show the full reaction of NDA-tagged dodecylamine and decylamine, respectively, for perspective with respect to retention time. (**I**) NDA-tagged amines (decylamine, dodecylamine, tetradecylamine, and octadecylamine). All elution profiles were isocratic with flow rates of 1 mL/min.

Ultra-trace detection of primary amines has been achieved by derivatization with fluorescent reagents. An attractive flourogen of choice is naphthalene-2,3-dicarboxaldehyde (NDA), due to commercial availability, inexpensive cost, and simple reaction schema [43,44]. At equimolar concentrations, varying the reactant (primary amine, potassium cyanide (KCN), NDA) ratios directly affects the fluorescence response. In examining reaction stoichiometries, the optimal reaction conditions of those tested in vitro at approximately 45 °C for 30 min was 1:20:24 (amine:KCN:NDA) (Figure 1). Given potential red and blue-shifting as a function of solvent polarity (relative to reported values), the emission wavelength was optimized based on our solvent conditions to maximize signal to noise ratio (S/N) as a function of emission wavelength. Using an excitation laser at 405 nm, the experimentally determined emission maxima (Em_max_) was approximately 470 nm (Figure 2). This Stokes shift of approximately 65 nm was wide enough to ensure that no reflected excitation radiation affected the background signal. To ensure proper fluorescent tagging of the primary amine and determine any native background fluorescence, all individual reactants and reaction combinations (maintaining 1:20:24 molar ratio, with methanol substituted for reactants not used) were examined using HPLC with fluorescence detection, as shown in Figure 3. An isocratic elution using 100% methanol and a reversed phase C_18_ column were used. In control studies, none of the individual reactants displayed any fluorescence response (Figure 3A–C), nor did binary mixtures of amine-NDA and amine-KCN reactions (Figure 3D,E, respectively). It should be noted that, although amine-NDA and amine-KCN showed no fluorescence response, the KCN-NDA binary mixture showed a relatively early eluting fluorescence peak, likely caused by dimerization of the NDA upon reaction with KCN. In the full reaction using a 12 and 10 carbon amine, the KCN-NDA dimerization peak was visible at 1.5 min at a much lower response as compared with the tagged amines (Figure 3G,H, respectively). A chromatogram of four fully saturated primary amines of acyl chain length varying from 10 to 16 carbons is shown in Figure 3I. Although baseline resolution could not be achieved between C10:0 and C12:0, the resolution between consecutive saturated amines did increase in accordance with increasing acyl chain length, with baseline resolution between C12:0 and C14:0 and all subsequent amines of increasing acyl chain length. It should be noted that biologically relevant amides typically contain acyl chain lengths of 16 or more carbons with varying degrees of unsaturation. Should co-elution occur between biologically relevant amines, mobile phase polarity can be reduced, or the use of a gradient elution profile can easily be employed. 

### 2.2. Segmented Flow Microfluidics

**Figure 4:**
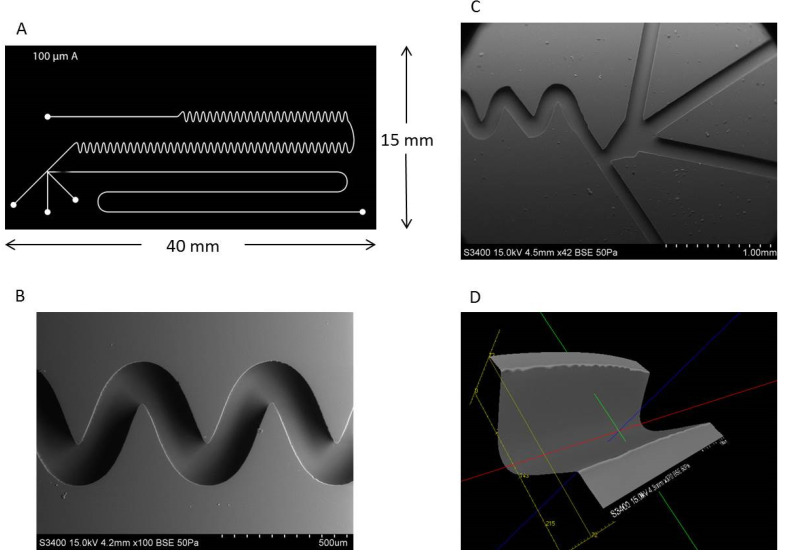
SEM imaging of channel features of a microfluidic device fabricated using SU-8 2075 photoresist as a mold master. (**A**) Cartoon schematic of final photomask used for polydimethylsiloxane (PDMS) microfluidic chip fabrication. (**B**) The four inlet channels, the T-junction, and the beginning of the mixing region are shown in a zoomed out image. (**C**) A zoomed in image of the mixing channel shows consistent curving features which allow for on-chip mixing and reactions. (**D**) The three dimensional image can be used to calculate distances between two points, enabling the determination of channel width and depth.

Droplet-based, or segmented flow, microfluidics is an advantageous technique to use in that it allows for highly efficient reactions, approximately 100-fold less solvent usage as compared to HPLC, and compatibility with highly sensitive photon detectors. The process of droplet formation has been reviewed and illustrated especially well by Song et al. [40]. The microchannel features were designed for specific application to fluorescent tagging of polar neutral lipids and are shown in detail in Figure 4. Figure 4A shows a graphical representation of the microchannel features with four inlet ports along the bottom of the chip and one outlet port at the top of the chip. The far left inlet flows an immiscible oil phase, perfluorodecalin, while the other three are designed for the reactants. The far right inlet has been elongated and includes a fritless weir near the junction point to allow for potential future chromatographic packing on-chip. Figure 4B,C show scanning electron microscope (SEM) images of the curved-channel mixing region and the droplet formation T-junction, respectively. All of the flow is pressure driven via external syringe pumps, with the immiscible phase flowing at a slightly higher flow rate than the sum of the reactants (all reactants’ flow rate is equal coming from a multi-port syringe pump). This difference in flow creates a droplet-producing taper effect where the reactants’ flow combines immediately prior to the junction, and the resulting droplets are carried through the channel by the immiscible phase. Due to the laminar nature of the flow through a straight channel, the droplets contain distinct layers representative of each reactant, with inefficient mixing occurring via simple diffusion between each intra-droplet fluidic layer. As the droplet passes through the curved region of the channel, it undergoes stretching, folding, and reorientation due to the sheer forces applied by the channel walls. Each turn in the channel doubles the fluidic layers to the point where they become increasingly thin, causing inter-layer diffusion to be an incredibly efficient form of mixing. The incident laser is focused centrally on the post-mixing region of the channel to fluoresce the sample after complete reaction has occurred. The completeness of the reaction was evaluated by collecting a fluorescence chronogram of droplet fluorescence from equimolar reactants flowing at identical rates. A previous channel design that incorporated only 10 turns in the mixing region resulted in inconsistent droplet fluorescence response (as discussed below). Of note, after optimization (see below) both the inter- and the intra-droplet variations in fluorescence response were observed when only 10 turns were included, indicative of incomplete and inconsistent reactions. The currently used channel design was modified to incorporate 140 turns in the mixing region. The improved reproducibility of fluorescence response maxima in each droplet using the modified chip design versus the previous chip design can be easily observed, signifying drastically improved on-chip fluorescent tagging reactions.

### 2.3. LIF Optical Detection System

**Figure 5:**
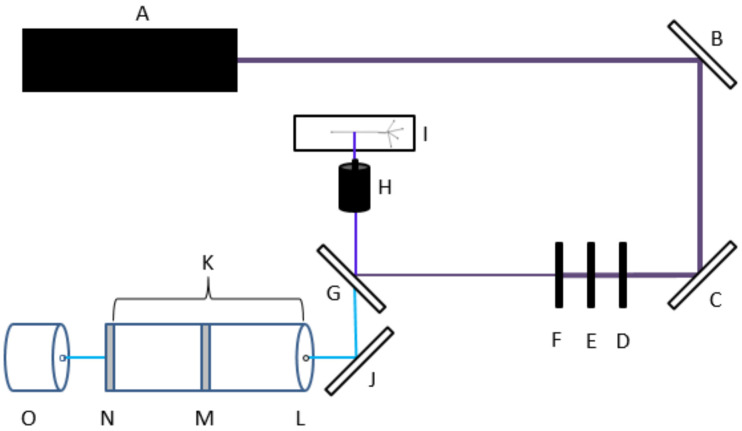
Schematic representation of the laser induced fluorescence (LIF) detection system. A 405 nm excitation laser beam was emitted from a solid-state laser (A) and reflected off of two mirrors (B,C). The beam then passed through a 405 nm emission bandpass filter (D), rotationally-mounted polarizer (E), and pinhole filter (F). The beam reflected off of a 425 nm dichroic lens (G) and focused onto the sample (I) using a microscope objective (H). The resonant fluorescence beam then passed back through the objective and the dichroic lens, then was reflected off of another mirror (J) and into the optics tube (K) through a pinhole filter (L). Inside of the tube was a 472 nm emission bandpass filter (M) and achromatic focusing lenses (N). The focused beam was detected by a single photon counting silicon avalanche photodiode array detector (APD) (O).

All optical components used in the LIF detection system must be meticulously arranged and aligned to ensure maximum signal to noise ratio (S/N). A representative schematic of the optical detection system is shown in Figure 5. The incident laser beam was produced from a vertically polarized diode-pumped solid state crystal laser at a wavelength of 405 nm. The beam was reflected off of a series of mirrors and passed through a 405 nm bandpass excitation filter. A pinhole filter removed the low-intensity laser scatter that occurred at the outer circumference of the beam. Our initial optical system utilized a rotation-mounted linear polarizer set to 300°, as this was expected to elicit maximum signal. However, further studies revealed an S/N increase of approximately 13% upon removing the polarizer altogether (unpublished observation). A longpass dichroic mirror with a cutoff wavelength of 425 nm was used to reflect incident excitation wavelength while allowing transmittance of red-shifted emission wavelength. A 40-fold magnification microscope objective lens was used to focus the laser beam onto the microchannel to excite the NDA-tagged amine sample. The resulting fluorescence emission at 472 nm passed back through the microscope objective and was transmitted through the dichroic lens. A final mirror reflected the fluorescence through a second pinhole filter and into dark optics tube, which contained a 472 nm emission bandpass filter as well as achromatic focusing lenses. The fluorescence emission beam was then focused onto the 180 µm diameter circular active area of a single photon counting module equipped with a silicon avalanche photodiode. Individual photons generated transistor-transistor logic pulses, which were binned over 25 ms increments and transmitted to data acquisition software.

**Figure 6:**
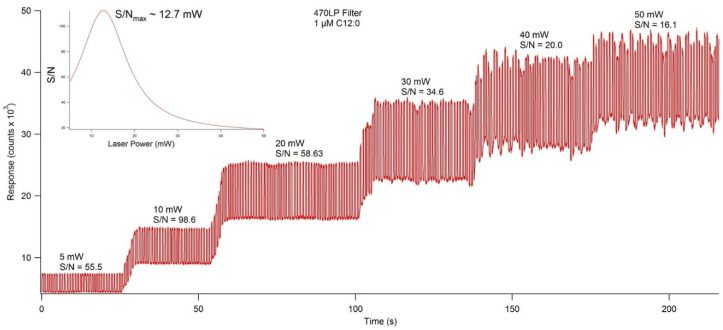
Fluorescence chronogram showing the effect on response and signal to noise ratio (S/N) caused by lasing power. Using the variable power controller, the lasing power was set to 5, 10, 20, 30, 40, and 50 mW, and fluorescence response was recorded at each increment. Of the individual power settings, 10 mW showed the highest S/N at 98.6, but extrapolation of the data fit to a Gaussian distribution showed the S/N_max_ to be at 12.7 mW (inset).

**Figure 7:**
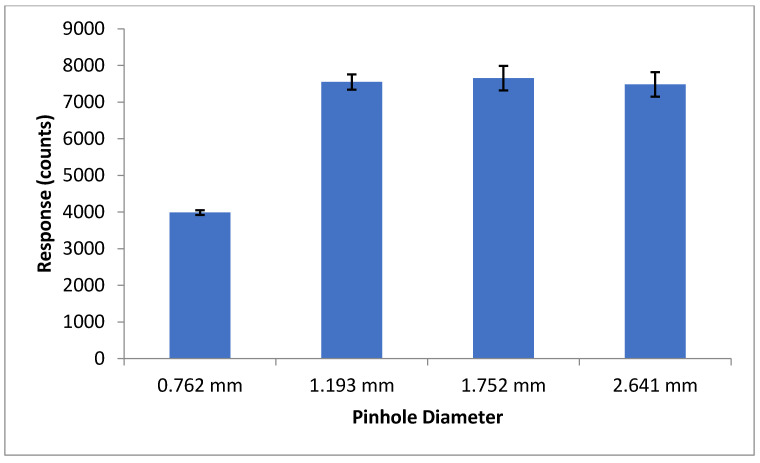
Average fluorescence response shown at each pinhole diameter setting. At a diameter of 0.762 mm, the average response was nearly half of that at 1.193 mm. Further opening the pinhole filter past 1.193 mm did not significantly increase the average response, but, given the increase in the error, appeared to increase the droplet-to-droplet variability (*n* = 7–10).

**Figure 8:**
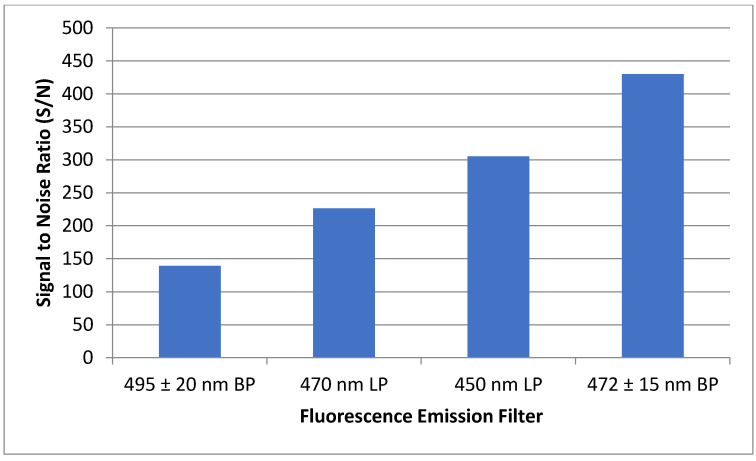
Four different emission filters were tested to determine maximum S/N of a 5 µM NDA-tagged hexadecylamine solution. The 472 nm bandpass filter showed the greatest S/N, with 450 nm longpass, 470 nm longpass, and 495 nm bandpass filters showing progressive decreasing of the S/N.

To ensure maximum sensitivity, several components of the optical detection system must be optimized. The concentration of fluorescent materials used in the optical detection system optimization experiments described in this section were arbitrarily selected with the crucial concern being that the fluorescence response is significant enough to be consistently detected. The variable power source allowed the user to control lasing power from 0–50 mW. Using NDA-tagged dodecylamine diluted to 1 μM, fluorescence chronograms were acquired using varying lasing power (Figure 6). As the lasing power was increased, both the response and the noise increased accordingly. By plotting the signal to noise ratio versus each respective lasing power, the optimal power setting was found to be 12.7 mW. The laser emitted an incident beam that approximated a Gaussian profile, with the center of the beam emitting the highest intensity radiation. As the distance from the center of the beam increased outward, the intensity was reduced, and variability increased. As shown in Figure 7, the response increased as the pinhole diameter increased. Once the approximate maximum response was reached, increasing the pinhole diameter only increased the noise. Using the maximum emission (Em_max_) wavelength of approximately 470 nm, several bandpass (BP) and longpass (LP) emission filters were tested using an NDA-tagged primary amine prepared similarly to the aforementioned. The calculated S/N from the corresponding fluorescence chronograms was determined for each filter and is shown in Figure 8. As expected, the 472 ± 15 nm BP filter presented the greatest S/N, as its maximum transmittance T_max_ was nearest to the Em_max_. Although the 450 nm LP filter recorded nearly three times the fluorescence response than the 472 BP filter, the greatly widened transmission band with the LP filters added more than four times the noise, resulting in a less sensitive optical detection system at ultra-trace concentration levels. Although both the 470 nm LP and the 495 ± 20 nm BP filters had transmission bands that extended to or near the calculated Em_max_, the percent transmittance at the Em_max_ was much lower than the T_max_, resulting in a lower S/N.

### 2.4. Droplet Formation and On-Chip Fluorescent Tagging

**Figure 9:**
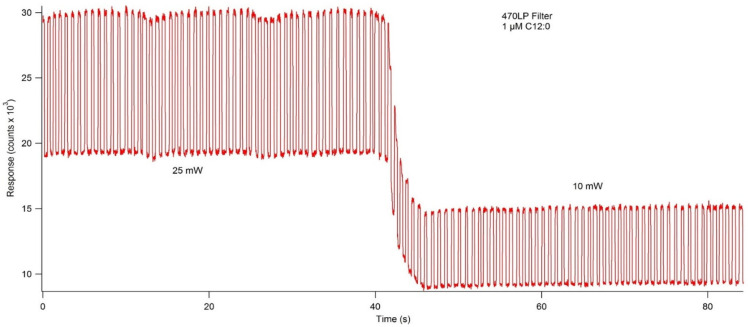
An example of a fluorescence chronogram showing consistent droplet formation. The lasing power was abruptly changed mid-acquisition and resulted in an immediate adjustment in response. Consistent droplet formation was determined by equal spacing between peaks (frequency) and equal peak widths (size).

Droplet-based microfluidics presents the ability to conduct highly efficient fluorescent tagging reactions on-chip, minimizing additional sample handling. For optimal rapid and consistent droplet formation, the flow rate of the immiscible oil phase must be greater than that of the reactant phase(s). It was found that a 4 µL/min flow rate of oil phase to 2 µL/min flow rate of the reactant phase(s) provided consistent droplet size and frequency, which can be observed by similar size and spacing of the peaks in a fluorescence chronogram (Figure 9). The droplet size can be inferred by the length of time that an individual peak of a fluorescence chronogram remains at its maxima, and frequency can be determined by the total number of peaks divided by unit time. It should be noted that, when multiple reactants are used for fluorescent tagging on-chip, the summation of their individual flow rates should equal the aforementioned 2 µL/min total reactant phase flow rate. The acquisition software was set to produce a binned data point every 25 ms, which was sufficient to record true maximum and minimum responses for each droplet.

**Figure 10:**
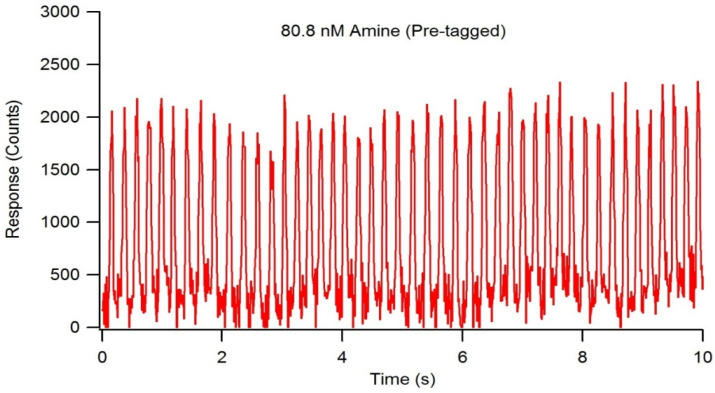
Fluorescence chronogram showing NDA-tagged decylamine approaching the detection limit of the LIF detection system. The tagging reaction occurred pre-chip, and the total amine concentration was serially diluted to 80.8 nM. The droplet frequency was determined to be 4.6 droplets/second, and average droplet volume was 5.4 nL/droplet, resulting in approximately 436 amol of amine per droplet.

Detection limit studies were performed using optimized LIF detection system settings, a reactant flow rate of 1.5 µL/min, and NDA-tagged dodecylamine serially diluted to an amine concentration of 81 nM. With a droplet frequency of 4.6 droplets/second, each droplet was calculated to contain 5.4 nL of solution. At an amine concentration of 81 nM, the average droplet contained 436 amol of fluorescently tagged amine with a signal to noise ratio of approximately 25 (Figure 10). Further dilutions to determine a more precise limit of detection were unsuccessful in obtaining a consistent, measurable fluorescence response, therefore, the actual detection limit was expected to be less than 436 amol.

**Figure 11:**
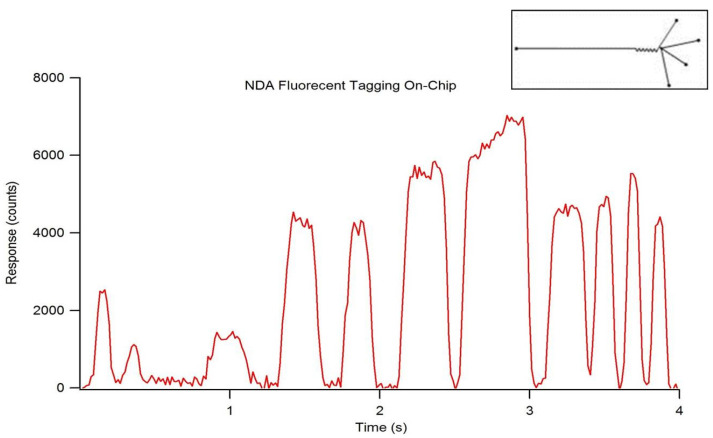
Fluorescence chronogram showing NDA-tagging reaction efficiency performed on-chip. Reactants were maintained in a 1:20:24 amine:KCN:NDA molar ratio and applied to the chip independently at 0.5 µL/min. Using a chip design with 10 turns in the mixing region (inset) resulted in an incomplete reaction, indicative of inconsistent maximum peak responses.

**Figure 12:**
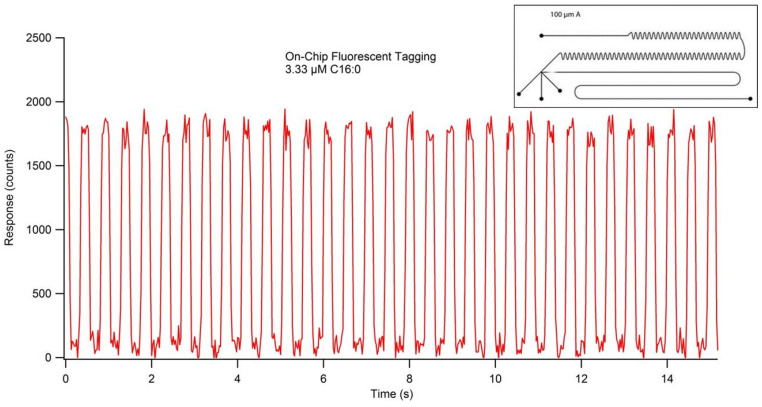
Fluorescence chronogram showing NDA-tagging reaction efficiency performed on-chip. Reactants were maintained in a 1:20:24 amine:KCN:NDA molar ratio and applied to the chip independently at 0.5 µL/min. Using a modified chip design with 130 turns in the mixing region (inset) resulted in complete reaction, indicative of consistent maximum peak responses.

On-chip NDA-tagging efficiency was determined using two different channel designs. A 1:20:24 (M/M/M) amine:KCN:NDA reaction concentration ratio was maintained with all reactants independently administered to the microchip at a flow rate of 0.5 µL/min (1.5 µL/min total flow). The resulting concentration of hexadecylamine per droplet was calculated to be 3.33 µM. The initial chip design contained a mixing region with 10 turns in the channel, which resulted in incomplete fluorescent tagging, as shown by inconsistencies in maximum droplet response by the fluorescence chronogram (Figure 11). Additionally, the varying peak widths indicate inconsistent droplet formation due to the presence of both droplets and elongated sample plugs. Transitioning to a new chip design containing 130 turns in the mixing region of the channel resulted in a completed fluorescent tagging reaction, as indicated by consistent maximum responses per droplet (Figure 12). Specific channel designs described herein present the capabilities of droplet formation, fluorescent tagging, and detection occurring in the order of seconds.

As shown in Figure 10, this microfluidic system demonstrated the capability of detecting concentrations of 436 amol per droplet at a signal to noise ration of approximately 25. Extrapolating the signal to noise ratio to a value of three, we estimated that, under these conditions, we could accurately detect concentrations down to 10^−17^ moles per droplet, which provides an improvement of up to three orders of magnitude over that of the currently existing HPLC-MS methodology (20 fmol limit of detection (LOD)). As discussed in the next section, this can provide an accurate quantification system of sufficient sensitivity to complement other analytical platforms such as mass spectrometry.

## 3. Discussion

The PDMS microfluidic device fabrication process using photolithography has numerous benefits inclusive of low cost per chip ($2 or less), relative safety (hydrofluoric acid required with glass chip fabrication process), rapid fabrication (approximately 1 day), and overall ease of use [45]. The key drawback to using PDMS is the relatively low pressure thresholds that induce the chip to unbond from the glass surface. When the PDMS is oxidized by air plasma inside of the chamber, hydrocarbons are etched, leaving behind surface silanol groups, resulting in a hydrophilic surface. When placed in contact with an oxidized glass surface, bridged siloxane bonds are formed at the interface, creating the seal [46]. This seal, however, has the tendency to fail with increasing pressures applied to the chip from external syringe or HPLC pumps. The application of an epoxy coating at the perimeter of the chip and the glass helps increase the pressure limits, however, there is still a tendency for relatively high pressures to force the fluid flowing through the channels to balloon outward, resulting in the chip failure and subsequent discarding.

During the photolithographic fabrication process, it is imperative to ensure a perfectly cleaned silicon wafer surface prior to spin coating. When conducting the soft bake process following UV exposure, the channel features begin to faintly appear in the photoresist. This is caused by the exposed region underneath the photomask to polymerize in the presence of UV light, resulting in this region being less soluble in the subsequent developing solution. Slight horizontal dispersion of the UV light through the photomask results in the channel width being slightly greater than that of the photomask. This is shown by the SEM micrograph having an average channel diameter of 155 µm, as opposed to the 150 µm width of the channels in the photomask. 

The LIF detection system requires precise alignment of all optical components in order to achieve fluorescence detection, with minute adjustments required for optimal S/N. It was determined that a greater S/N was achieved by complete removal of the rotation-mounted polarizer. This was somewhat expected when results showed the greatest response to be in a vertically polarized position, which is the native polarization of the incident laser. The increase in response by removing the polarizer was due to the percent transmittance of the polarizer being less than 100%. In optimizing the pinhole filter diameter, the fluorescence response increased as the diameter of the pinhole filter was increased, up to 1.193 mm, at which point no further response gain was observed. As the filter was opened past 1.193 mm, a slight increase in noise was noticed while the signal remained constant. The goal of the pinhole filter is to block the lower, more variable outer regions of the laser beam. A cross-sectional view of the laser beam would show a Gaussian distribution in laser intensity, with the center of the laser having the greatest intensity and the outer regions showing higher variability. The dichroic lens displays an important function in that it acts as an additional filter, preventing the incident laser wavelength from reaching the detector. At a 425 nm longpass cutoff, the dichroic lens reflects all light with a wavelength less than 425 nm (incident laser wavelength = 405 nm) and transmits all light with a wavelength greater than or equal to 425 nm (emission maxima = 470). Of the four emission filters tested, the 472 nm bandpass filter provided the highest S/N, as it falls in line with the optimal emission maxima of NDA-tagged amines. A significant detector to detector response variability existed between the three detectors tested, with the Perkin Elmer avalanche photodiode array detector (APD) displaying a significantly greater response as compared with the two EG&G APDs. The positioning of the microchip on the sample stage is crucial to achieving a maximum S/N. To prevent misalignment caused by involuntary movement of the chip, it is held in place using tape, and the entire stage is carefully adjusted in X, Y, and Z planes. Optimal positioning of the focused laser beam should be in the center of the detection region of the channel, and the bottom of the glass should be within 1 mm of the focusing microscope objective. Meticulous vertical alignment of the stage in relation to the microscope objective is required while monitoring real-time fluorescence response data until the greatest signal is achieved.

**Figure 13:**
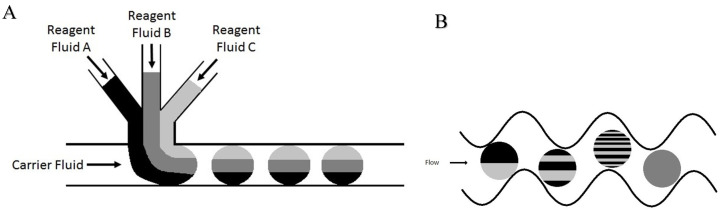
Illustration of droplet formation within microfluidic devices using a T-junction and mixing. (**A**) The use of a standard T-junction introduces the reagent fluid to the carrier fluid in an orthogonal manner. When the carrier fluid flow rate is greater than that of the sum of all of the reagent fluids, the reagent fluids taper off into individual droplets. (**B**) Process of chaotic advection within droplets in a mixing region of a microfluidic device. Beginning with a droplet containing two discrete fluidic layers, the frictional forces induced on the droplet by the channel walls cause continuous refolding and recirculation of the droplet. As mixing within droplets is primarily based on inter-layer diffusion, the multiplication and the subsequent thinning of fluidic layers result in highly efficient mixing within droplets.

When optimizing flow rates for consistent droplet formation, it is necessary to have the immiscible oil phase be greater than that of the reactant(s) phase. The oil phase acts as a carrier fluid, with the reactants combining at the T-junction and tapering off into small volume droplets, which are carried down-channel by the immiscible oil (Figure 13A). Due to the extremely low flow rates and the relatively long length of tubing, a significant amount of time is usually required for stable flow to occur, resulting in consistent droplet formation. The on-chip NDA-tagging reactions shown in Figure 11 and Figure 12 illustrate the importance of sufficient mixing. A linear channel exhibits laminar flow, with the only mixing occurring by simple inter-layer diffusion. By introducing the turns into the channel design, reorientation and folding of the fluidic layers within the individual droplets, known as chaotic advection, are induced [38,39,40]. Each turn provides an additional fluidic layer interface, with subsequent turns rendering this fluidic layer infinitesimally thin to the point where simple diffusion becomes a fast and efficient form of mixing (Figure 13B). Initial chip designs incorporating 10 turns into the mixing region proved insufficient, as shown by the resulting fluorescence chronogram. The varying peak widths found in Figure 11 were the result of unstable droplet formation, not that of incomplete reaction. The varying maximal response peak to peak shows that certain droplets exhibited greater reaction efficiency than others. Additionally, broad peaks (indicative of sample plugs rather than droplets) such as the one at approximately 2.75 min showed a fluorescence response increasing within the individual droplet. This shows that the reaction efficiency at the front end of the droplet was worse than the efficiency at the tail end. Reaction efficiency was shown to be much improved using the modified chip design that incorporated 130 turns in the mixing region (Figure 12) due to longer residence time of the droplet components within the mixing region of the chip. The consistent maximal response within and between droplets indicated that the reaction was complete. 

The use of microfluidics with LIF was developed to provide the potential to rapidly tag and detect ultra-trace quantities of bioactive lipids for use in lipidomics studies via fluorescence detection. This NDA chemical tagging schema may also be readily adapted for any amines and can have utility in metabolomics and proteomics studies. This is especially important in discovery studies, where typical quantification methodologies used in MS-based studies involving isotope enrichment are not feasible. To be a useful adjunct, the LIF system must have sensitivity that rivals or is of greater sensitivity than that of mass spectrometry platforms. Detection limits studies using this microfluidic device and LIF analysis system had detection capabilities at 436 amol per droplet with a signal to noise ratio of approximately 25. We estimate that, under these conditions, we could accurately detect concentrations down to 10^−17^ moles per droplet, which provides an improvement of up to three orders of magnitude over that of the currently existing HPLC-MS methodology (20 fmol LOD) [47]. As LIF is a non-destructive detection technique, microfluidic device fabrication can be further modified to incorporate seamless integration with MS detection. Using this proposed analysis system design, LIF can be used for highly sensitive quantification, while parallel MS or other analytical methods can provide accurate identification of the tagged species. Of note, parallel complementary quantitation may be easily adapted for any amine and may be useful in proteomics-based as well a lipidomics-based studies.

## 4. Materials and Methods 

### 4.1. Chemicals

Methanol was purchased from Fisher Scientific (Fair Lawn, NJ, USA). Toluene, decylamine (C10:0), dodecylamine (C12:0), and octadecylamine (C18:0) were purchased from Sigma Aldrich (St. Louis, MO, USA). Potassium cyanide (KCN), chlorotrimethylsilane, tetradecylamine (C14:0), hexadecylamine (C16:0), and oleylamine (C18:1^9^) were purchased from Acros Organics (Bridgewater, NJ, USA). The 3-(2-furoyl)quinoline-2-carboxaldehyde (FQCA) and the naphthalene-2,3-dicarboxaldehyde (NDA) were purchased from Molecular Probes (Eugene, OR, USA). Perfluorodecalin (PFD) was purchased from Alfa Aesar (Ward Hill, MA, USA). SU-8 2075 positive epoxy photoresist and SU-8 developer were purchased from MicroChem (Newton, MA, USA). Nano-Strip resist remover was purchased from Cyantek (Fremont, CA, USA). Sylgard 184 silicone elastomer kit was purchased from Dow Corning (Midland, MI, USA). 

### 4.2. Instrumentation

Microchannel templates were designed using a vector graphics editor (Adobe Illustrator CC, Adobe Systems, Mountain View, CA, USA), and chrome masks were fabricated via Front Range Photomask (Palmer Lake, CO, USA). Uniform resist coating was applied to silicon wafers using a universal spin processor (WS-650 Spin Coater, Laurell Technologies, North Wales, PA, USA), and photopolymerization of the resist was performed using a 500 W near-UV mercury arc lamp (Newport, Model 97435-1000-1, Newport Corporation, Irvine, CA, USA). Hydrophilic rendering and oxidation of surfaces of finished PDMS microchips and glass bases were performed using air-plasma treatment (PCD-32G, Harrick Plasma, Ithaca, NY, USA). Fluorescence excitation was performed using a variable power 50 mW 405 nm blue laser (DL-405-050, CrystaLaser, Reno, NV, USA) and emission detected using a single photon counting module (PerkinElmer SPCM-AQR-14, Excelitas Technologies, Waltham, MA, USA). All optical filters and components were purchased from Omega Optical (Brattleboro, VT, USA), Edmund Optics (Barrington, NJ, USA), and ThorLabs (Newton, NJ, USA). Pre-chip chromatographic separations were performed using a high performance liquid chromatograph (Ultimate 3000, Dionex Corporation, Sunnyvale, CA, USA) and microbore C_18_ column (XTerra, 5 µm, 0.32 × 50 mm, Waters Corporation, Pittsburgh, PA, USA). All data were collected and processed using graphical programming software (LabView 2012, National Instruments, Austin, TX, USA). 

### 4.3. Fluorescent Labeling of Amines

For optimal fluorescent tagging of amines using FQCA, 10 mM solutions of both FQCA and amine were prepared in methanol, and a 10 mM solution of KCN was prepared in water. Optimal reaction conditions were determined to be 1:10:12 (*v*/*v*/*v*) equimolar amine:KCN:FQCA. The reactants were mixed in an amber glass vial and allowed to react for approximately 45 min at 40 °C using a heating block. Fluorescent tagging of amines using NDA follows a similar procedure to that of FQCA. Each reactant stock solution was prepared at 10 mM with both NDA and amine in methanol and KCN in water. The reactants were mixed in an amber glass vial and heated at 40 °C for approximately 30 min for the reaction to proceed to completion. 

### 4.4. Reversed Phase HPLC Separation of Primary Fatty Acid Amines

A Waters 1525 Binary HPLC Pump with Waters 2475 Multi Wavelength Fluorescence Detector and Waters Breeze version 3.30 software package was used for separation and fluorescence detection of amine mixtures. A Waters Xterra MS C18 with 5 um particle size, 3.9 mm × 150 mm column was used to separate saturated fluorescently tagged amine standards. A 5 uL injection loop was used for consistent injection volumes, and the column was maintained at room temperature. An isocratic elution profile consisting of 100% methanol with a flow rate of 1.5 mL/min was used. For analysis of FQCA-tagged amines, an excitation wavelength of 488 nm and an emission wavelength of 590 nm were used with gain and energy units full scale (EUFS) settings at 100 and 1000, respectively. For analysis of NDA-tagged amines, an excitation wavelength of 405 nm and an emission wavelength of 470 nm were used with gain and EUFS settings at 100 and 1000, respectively. 

### 4.5. Microchip Fabrication

Microchannel designs were patterned onto chrome coated soda lime glass at a resolution of ±0.5 µm to yield a 6 × 6 inch circular photomask. Approximately 5 mL of SU-8 2075 photoresist was applied to the center of a 150 mm silicon wafer and spin coated to a uniform thickness of approximately 108 µm, which translates to the channel depth in the fabricated microchip. A spin coater was programmed to ramp to 500 rpm for 10 s followed by a second ramp to 2000 rpm for 30 s. The resist-coated wafer was then soft-baked at 65 °C for 5 min, returned to room temperature, and then heated to 95 °C for 25 min. UV exposure of the resist-coated wafer through the photomask was performed for 30 s at a distance of 20 cm and resulted in visible channel designs appearing in the resist. The wafer underwent a post-exposure bake at 65 °C for 5 min, returned to room temperature, and was again heated to 95 °C for 12 min. The wafer was developed for 10 min to remove all un-exposed resist and was rinsed with additional fresh developer, isopropyl alcohol, and deionized water and dried under a stream of nitrogen gas. At this point, an optional hard bake curing could be performed at 200 °C for 15 min, however, this was deemed unnecessary as the positive resist channel designs were firmly deposited on the wafer. 

The PDMS was prepared by mixing the silicon elastomer base and the curing agent in a 10:1 (m/m), respectively. The mixture was then desiccated to remove all air bubbles that formed during mixing. The PDMS was carefully poured over the master to ensure that there were no air bubbles on the surface and was cured for at least 1 h at 100 °C. After curing, the PDMS polymer was gently peeled from the master, and the inlet ports were punched at their designated locations. After being thoroughly cleaned using isopropyl alcohol and dried, the PDMS microchip and the glass base were plasma cleaned for 3–5 min at high power (30 W). Once removed, the substrates were applied to one another to form a spontaneous seal. To ensure the seal under pressure driven flow, quick-setting epoxy was applied to the perimeter of the PDMS chip and the glass base. Polyetheretherketone tubing was inserted into each port, and epoxy was used to provide a leak-proof seal. 

### 4.6. Scanning Electron Microscopy

A Hitachi S-3400N scanning electron microscope (SEM) was used for analysis and verification of microfluidic device features. Prior to bonding to glass, a clean microfluidic chip was positioned feature side up on a 15 mm aluminum sample stub affixed to a copper stub holder. The high voltage was turned off, and the SEM chamber was exposed to air prior to opening. Once opened, the stub holder, the stub, and the sample were placed onto the specimen stage, and the total height was measured to ensure no contact was made with the metal optics guard. This height was inputted into the software program, and the stage height was adjusted accordingly (sample should be approximately 1 mm from the optics guard). The chamber was closed, and air was evacuated with a vacuum pump until internal pressure reached 50 Pa. With an accelerating voltage of 15.0 kV, the sample image was brought into focus by adjusting magnification and focus knobs on the external controller. Brightness and contrast settings were also adjusted to provide the sharpest image. Both two- and three-dimensional images of the microfluidic device were acquired.

### 4.7. Development of a Laser Induced Fluorescence Detection System

For the development of an LIF detection system, all optical components were purchased from ThorLabs. The excitation source was a CrystaLaser Violet Blue solid-state laser with a wavelength of 405 nm (±5 nm), variable power source from 0–50 mW, and vertical polarization. The incident beam was passed through a 405 nm bandpass excitation filter, a rotation-mounted linear polarizer set to 300°, and a pinhole filter. A longpass dichroic lens with cutoff wavelength of 425 nm reflected incident laser light vertically through a 40-fold magnification microscope objective lens, focusing the beam to the desired detection region of the microfluidic device. The resulting fluorescence emission in the downward vertical plane passed back through the microscope objective and the dichroic lens to a final mirror to reflect the fluorescence light horizontally to an optics tube. The optics tube contained a pinhole filter at the front end with a 472 nm emission bandpass filter and achromatic focusing lens within. The fluorescence emission beam was focused onto the 180 µm diameter circular active area of a single photon counting module equipped with a silicon APD. Individual photons generated transistor–transistor logic pulses, which were binned over 25 ms increments and transmitted to LabView data acquisition software. 

Preliminary LIF detection system alignment was conducted by visually aligning the excitation laser beam downstream from component to component. In lieu of a fluorescing sample being placed on the horizontal sample stage above the focusing microscope objective, a mirror was used to reflect the laser beam back to the center of the dichroic lens. Alignment of the optical components downstream from the second passing of the dichroic lens relied on a concentrated NDA-tagged dodecylamine solution loaded into a capped, flat-sided quartz cuvette placed on the sample stage. The resonant fluorescence beam was aligned through the dichroic lens and reflected into an optics tube. Additional lenses within the optics tube focused the beam onto the active area of the photon counting detector. Finally, a microfluidic chip undergoing segmented flow with NDA-tagged dodecylamine was placed on the sample stage and the stage aligned until the excitation laser beam was focused directly within a sample channel. The chip was taped to the sample stage during the alignment process to ensure no involuntary movements of the chip would cause subsequent misalignment. The sample stage as well as the optical components downstream were adjusted until the detected fluorescence droplet response achieved the greatest S/N. All alignment procedures were performed in complete darkness to allow for maximum excitation and emission beam visibility.

Several optical components required optimization to achieve the greatest possible S/N of NDA-tagged amine. Signal was recorded while adjusting a pinhole filter from closed to its maximum diameter of approximately 2.6 mm, and precise diameters were measured using a caliper. The laser polarization was adjusted from vertical to horizontal alignment, and the rotation-mounted polarizer was removed from the beam path. Several emission filters inclusive of 495 nm and 472 nm bandpass filters and 470 nm and 450 nm longpass filters were tested. Using the variable power controller, the lasing power was tested between 5 mW and 50 mW. To determine variance caused by specific APDs, a Perkin Elmer APD was tested against two different models of EG&G APDs. On-chip fluorescent tagging efficiency was examined using two different channel designs; one design contained a droplet mixing region containing 10 turns before the linear detection channel, and a second design contained 130 turns before the detection channel. A preliminary investigation of pre-chip chromatographic separation was conducted using a Dionex Ultimate 3000 HPLC Pump with a Waters Xterra MS C18 column (5 µm, 3.9 mm × 150 mm). The flow rate was held constant at 4 µL/min, and a 2 µL injection volume was used. 

### 4.8. Droplet Based Microfluidic Flow

The formation of droplet-based flow within the microfluidic device is crucial for conducting on-chip reactions. To optimize droplet formation flow rates, only two inlet ports were used. The inlet port with the channel that continues straight past the T-junction was used for the flow of perfluorodecalin, an immiscible oil phase. One of the three inlet ports with channels entering the T-junction (perpendicular to the oil phase channel) was used for the flow of NDA-tagged dodecylamine. Perfluorodecalin and the tagged amine solution were loaded into separate syringes and applied to the microchip using independent syringe pumps. Flow rates were adjusted until rapid and consistent droplets were formed on-chip. Droplet formation was detected visually due to differences in light refraction properties of perfluorodecalin and methanol as well as by fluorescence detection. 

To conduct on-chip NDA tagging reactions, a microchip was used with all four inlet ports accessible with PEEK tubing. The oil phase inlet was maintained as previously described, with the other three inlets used for the three reactants in the NDA tagging reaction. Amine, KCN, and NDA solutions were prepared at a concentration ratio of 1:20:24, respectively, and each was loaded into a syringe. The three reactant syringes were loaded onto a multi-syringe pump and all applied to the microchip simultaneously at the same flow rate. Flow rates were optimized for rapid and consistent droplet formation.

## 5. Conclusions

The ability to conduct fluorescent tagging reactions and the incorporation of chromatographic separations on-chip enable the possibility of a true µTAS system of analysis, dramatically reducing any errors caused by sample handling. Microfluidics also presents the opportunity to move towards a green analysis system, with a dramatic reduction of chemical and sample usage due to the extremely low flow rates and the volumes on-chip. Further work towards optimizing on-chip chromatographic separation efficiency by addressing the inherent PDMS robustness issues will be required prior to the investigation of using a microfluidic system for analysis and highly sensitive detection of biologically endogenous PFAMs.

## Data Availability

The data presented in this study are available on request from the corresponding author.
